# Protein-Remodeling Factors As Potential Therapeutics for Neurodegenerative Disease

**DOI:** 10.3389/fnins.2017.00099

**Published:** 2017-02-28

**Authors:** Meredith E. Jackrel, James Shorter

**Affiliations:** Department of Biochemistry and Biophysics, Perelman School of Medicine at the University of PennsylvaniaPhiladelphia, PA, USA

**Keywords:** protein-remodeling factors, protein-misfolding disease, neurodegeneration, Hsp104, Hsp70, Hsp110, NMNAT, HtrA1

## Abstract

Protein misfolding is implicated in numerous neurodegenerative disorders including amyotrophic lateral sclerosis, Parkinson's disease, Alzheimer's disease, and Huntington's disease. A unifying feature of patients with these disorders is the accumulation of deposits comprised of misfolded protein. Aberrant protein folding can cause toxicity through a loss or gain of protein function, or both. An intriguing therapeutic approach to counter these disorders is the application of protein-remodeling factors to resolve these misfolded conformers and return the proteins to their native fold and function. Here, we describe the application of protein-remodeling factors to alleviate protein misfolding in neurodegenerative disease. We focus on Hsp104, Hsp110/Hsp70/Hsp40, NMNAT, and HtrA1, which can prevent and reverse protein aggregation. While many of these protein-remodeling systems are highly promising, their activity can be limited. Thus, engineering protein-remodeling factors to enhance their activity could be therapeutically valuable. Indeed, engineered Hsp104 variants suppress neurodegeneration in animal models, which opens the way to novel therapeutics and mechanistic probes to help understand neurodegenerative disease.

## Introduction

There are numerous devastating, and incurable, neurodegenerative disorders that are increasing in prevalence as our population ages (Dobson, 2003; Forman et al., 2004; Morimoto, 2006). These disorders include: Alzheimer's disease (AD), Parkinson's disease (PD), amyotrophic lateral sclerosis (ALS), and frontotemporal dementia (FTD) (Dobson, 2003; Forman et al., 2004; Morimoto, 2006; Lagier-Tourenne et al., 2010; Robberecht and Philips, 2013). Treatments for these disorders remain palliative, and no therapeutics are available that address their underlying cause (Forman et al., 2004; Robberecht and Philips, 2013). Furthermore, each of these disorders manifests in different ways in patients. For instance, AD patients have impaired memory yet their movement is preserved, while ALS patients' memory is preserved while their control of movement becomes impaired (Forman et al., 2004; Lagier-Tourenne et al., 2010; Robberecht and Philips, 2013). Yet, at the fundamental level, these neurodegenerative disorders are linked by the presence of insoluble proteinaceous inclusions in the brain (Dobson, 2003; Forman et al., 2004; Lagier-Tourenne et al., 2010; Robberecht and Philips, 2013).

It is important to note that these neurodegenerative diseases are not due to mass protein misfolding, but instead the misfolding of specific proteins are implicated in each disease (Dobson, 2003; Cushman et al., 2010). For instance, α-synuclein misfolds into amyloid fibrils that accumulate in Lewy bodies in the dopamine neurons of PD patients, while in ALS patients TDP-43 or FUS misfold into cytoplasmic aggregates in degenerating motor neurons and glia (Spillantini et al., 1997; Neumann et al., 2006; Chen-Plotkin et al., 2010; Mackenzie et al., 2010; Robberecht and Philips, 2013; Dehay et al., 2015). These proteins, as well as many others that underpin diverse neurodegenerative disorders, are expressed in nearly all cells. Yet it remains perplexing what initiates and drives the misfolding of specific proteins in specific neuronal subtypes, leading to subtype-specific neurodegeneration (Saxena and Caroni, 2011). Additionally, it remains unclear if neuronal degeneration is always a direct consequence of aggregate accumulation. Indeed, many of these proteins serve essential functions, and so a loss of function due to aggregation could alternatively lead to toxicity (Winklhofer et al., 2008; Yang et al., 2014; O'Rourke et al., 2016).

In each of these neurodegenerative disorders, the protein homeostasis (proteostasis) network ultimately fails to combat the accumulation of misfolded conformers, consequently leading to disease (Balch et al., 2008; Shorter, 2016). To address the protein-misfolding problem, there are several avenues that could be explored. First, degradation of the toxic, misfolded conformers might be beneficial. For instance, in some PD patients, an increase in α-synuclein levels is implicated, and thus degradation of this excess α-synuclein might be beneficial (Ebrahimi-Fakhari et al., 2012). A similar strategy might be useful in Huntington's disease patients (Yamamoto et al., 2000). Alternatively, stalling the protein-misfolding process is an effective means of therapeutically treating patients with familial amyloid neuropathy (FAP) (Bulawa et al., 2012; Cho et al., 2015; Ankarcrona et al., 2016). FAP is caused by the misfolding of transthyretin, which forms amyloid fibrils that accumulate in various tissues and organs, ultimately leading to organ failure. To combat FAP, the drug Tafamidis was developed to stabilize the native tetrameric form of transthyretin, thus blocking further misfolding and stalling the amyloid cascade. Tafamidis is approved for use by the European Medicines Agency, and is the only therapeutic in use that mitigates neurodegenerative disease by preventing protein misfolding (Ruberg and Berk, 2012). Additionally the drug Tolcapone, which is FDA-approved for PD, was found to also stabilize transthyretin and block aggregation (Sant'Anna et al., 2016). A similar strategy to pharmacologically stabilize α-crystallins may effectively block their misfolding and aggregation and treat cataracts (Makley et al., 2015). The success of Tafamidis provides strong proof of concept that targeting protein misfolding can be therapeutically effective (Bulawa et al., 2012; Cho et al., 2015; Ankarcrona et al., 2016). Additionally, clinical trials are ongoing to assess the efficacy of antibodies aimed at clearing plaques comprised of Aβ that accumulate in AD patients (Sevigny et al., 2016), though notably one trial recently failed. Indeed, an additional intriguing possibility would be to remodel the misfolded species such that the protein regains its functional, native conformation, which would simultaneously mitigate toxicity due to loss-of-function or gain-of-function (Jackrel and Shorter, 2014b, 2015; Mack and Shorter, 2016; Shorter, 2016). However, many of the proteins that misfold in these disorders adopt a cross-beta fibrillar form, termed amyloid, which is a highly stable and self-templating structure (Dobson, 2003). Nonetheless, protein-remodeling factors that have evolved to antagonize protein misfolding could be harnessed to reverse deleterious protein misfolding in disease (Table 1).

**Table 1:** **Protein-remodeling factors can remodel diverse substrates**.

**Protein remodeling factor**	**Activity**	**Substrates remodeled**
Hsp70	Blocks misfolding	Polyglutamine, α-syn, Aβ
Hsp110/Hsp70/Hsp40	Dissolves preformed aggregates	SOD1, α-syn
NMNAT	Dissolves preformed aggregates	Tau
Htra1	Dissolves and degrades preformed aggregates	Aβ and tau
Hsp104	Dissolves preformed aggregates, amyloid, and pre-amyloid oligomers	α-syn, TDP-43, FUS, Aβ, tau, polyglutamine

The proteostasis network ultimately collapses in neurodegenerative disease (Shorter, 2016). This network is comprised of many molecular chaperones that normally promote the proper folding of disease-associated proteins, as well as the entire proteome. Thus, an intriguing way to address the collapse of the proteostasis network would be to remedy or rewire this network (Jackrel et al., 2014a; Jackrel and Shorter, 2014b, 2015). This approach could be pursued by either enhancing and tuning the activity of endogenously expressed protein-remodeling factors, or by introducing new protein-remodeling factors that are not normally expressed (Warrick et al., 1999; Auluck et al., 2002; Jackrel et al., 2014a). Many protein-remodeling factors have been proposed to function in alleviating protein misfolding, including: Hsp104, Hsp110/Hsp70/Hsp40, NMNAT, and HtrA1 (Zhai et al., 2008; Jackrel and Shorter, 2014b, 2015; Poepsel et al., 2015; Ali et al., 2016; Mack and Shorter, 2016; Shorter, 2016). Some of these proteins are capable of actively disaggregating and restoring the solubility of the misfolded conformers (Warrick et al., 1999; Auluck et al., 2002; Jackrel and Shorter, 2014a; Jackrel et al., 2014a). Thus, the application of protein-remodeling factors in a therapeutic setting is a highly promising avenue to address neurodegenerative disease. In this review, we discuss the potential application of these molecular chaperones and protein disaggregases in the development of therapeutics for neurodegenerative disorders. These agents might be harnessed for therapeutic purposes through upregulation or through the introduction of exogenous protein through either gene therapy using adeno-associated viral vector technologies or direct injection. Alternatively, protein-remodeling factors could be therapeutically modulated using small molecules or even potentiated via engineering. We focus on efforts to reformulate a robust protein disaggregase from yeast, Hsp104, which has several unique properties that make it a particularly promising protein-remodeling factor for further exploration and application to reverse the protein misfolding implicated in numerous devastating neurodegenerative diseases (Lo Bianco et al., 2008; DeSantis et al., 2012; Cushman-Nick et al., 2013; Jackrel and Shorter, 2014a,b, 2015; Jackrel et al., 2014a). We also discuss several other protein-remodeling factors that have been recently assessed for their capacity to suppress or reverse protein misfolding connected to neurodegenerative disease.

## Hsp70 blocks protein misfolding

One of the first molecular chaperones to be explored as a possible therapeutic for combating neurodegenerative disease was Hsp70. The Hsp70 family of proteins serves diverse functions in protein folding. Hsp70 promotes the refolding of aggregated or misfolded proteins (Mayer and Bukau, 2005; Mack and Shorter, 2016). It also serves to ensure the proper folding of newly synthesized proteins (Mayer and Bukau, 2005). To do so, Hsp70 functions cooperatively with its co-chaperone, Hsp40, to bind and thus protect hydrophobic stretches harbored by its clients (Mayer and Bukau, 2005; Mashaghi et al., 2016). This function is crucial during protein synthesis, but is also important following cellular stresses that partially denature mature proteins, because by binding exposed stretches on these partially denatured proteins, Hsp70 can block protein aggregation (Mayer and Bukau, 2005; Mack and Shorter, 2016). Thus, in disease, upregulation of Hsp70 might prevent protein aggregation and promote the restoration of proteostasis. A *Drosophila* model of polyglutamine misfolding has been established in which overexpression of polyglutamine leads to neurodegeneration (Warrick et al., 1998). In this model, overexpression of Hsp70 suppressed polyglutamine-induced neurodegeneration (Warrick et al., 1999). Similarly, in a *Drosophila* model of α-synuclein misfolding, Hsp70 suppressed neurodegeneration (Auluck et al., 2002). However, it is important to note that while Hsp70 inhibited neurodegeneration in these models, it was not found to solubilize aggregates (Warrick et al., 1999; Auluck et al., 2002; Cushman-Nick et al., 2013). Nonetheless, in a mouse model of ALS, intraperitoneal injection of human Hsp70 increased lifespan, delayed the onset of symptoms, arrested denervation, preserved axonal function, and prolonged motor neuron viability (Gifondorwa et al., 2007, 2012).

Elevating Hsp70 expression can slow neurodegeneration in fly and mouse models (Warrick et al., 1999; Auluck et al., 2002; Gifondorwa et al., 2007, 2012). Hsp70 likely becomes overwhelmed in neurodegenerative disease. Thus, it may be important to enhance Hsp70 activity via potentiating mutations or small molecules (Mack and Shorter, 2016; Shorter, 2016). Indeed, using protein-engineering techniques the activity of the bacterial homolog of Hsp70, DnaK, has been enhanced and these variants demonstrate elevated luciferase refolding activity (Aponte et al., 2010; Schweizer et al., 2011). Recently, Hsp70 engineering has been extended to human Hsp70 and neurodegenerative disease-associated substrates (Aprile et al., 2015). Here, Hsp70 was tuned through rational design to more potently bind α-synuclein and Aβ42. Peptides complementary to target epitopes in α-synuclein and Aβ42 were developed, and these peptides were introduced into the C-terminal region of Hsp70 (Aprile et al., 2015). While introduction of these peptides enhanced the binding affinity of Hsp70 to α-synuclein and Aβ42, binding to other client proteins was unaffected (Aprile et al., 2015). Thus, tuning Hsp70 to broaden its substrate specificity does not come at the cost of restricted capacity to regulate its diverse client pool (Aprile et al., 2015). Additionally, small molecules have been identified that can enhance specific aspects of Hsp70 activity. For instance, four small molecules: MKT-077, JG-98, YM-1, and YM-8 bind the nucleotide-binding domain of Hsp70 in the ADP, but not ATP-bound state. This binding stabilizes the ADP-bound state resulting in increased affinity of Hsp70 for its clients, which can under some circumstances lead to their enhanced folding (Rousaki et al., 2011; Miyata et al., 2013; Wang et al., 2013; Shorter, 2016). In the cellular environment, YM-1 promotes clearance of polyglutamine oligomers and aggregates (Wang et al., 2013). All four of these molecules promote the clearance of tau and are therapeutically beneficial in tauopathy models (Abisambra et al., 2013; Miyata et al., 2013; Fontaine et al., 2015).

## The metazoan protein-disaggregase system: Hsp110/Hsp70/Hsp40

It has long been hypothesized that humans might possess a protein disaggregase similar to those in the Hsp100 family of proteins that are highly conserved in bacteria, fungi, and plants (Shorter, 2008, 2011; Torrente and Shorter, 2013). However, the discovery of such a protein disaggregase has been elusive until it was discovered that Hsp110 in collaboration with Hsp70 and Hsp40 can disaggregate and reactivate protein (Shorter, 2011; Mattoo et al., 2013; Torrente and Shorter, 2013; Finka et al., 2015; Gao et al., 2015; Nillegoda and Bukau, 2015; Nillegoda et al., 2015). Hsp110 is an Hsp70 family member that in collaboration with Hsp70 and Hsp40 can disaggregate preformed aggregates and amyloid (Shorter, 2011; Duennwald et al., 2012; Gao et al., 2015; Nillegoda et al., 2015). Hsp110 collaborates and synergizes with Hsp70 and two classes of Hsp40 cochaperones to resolve large protein aggregates (Nillegoda and Bukau, 2015; Nillegoda et al., 2015). It is hypothesized that due to the large number of possible complexes that could form between different Hsp70s and Hsp40s, distinct and specific complexes might be harnessed to dissolve different protein aggregates (Nillegoda and Bukau, 2015; Nillegoda et al., 2015). Perhaps one specific combination might be employed in specific neuronal subtypes, or a given combination might specifically disaggregate α-synuclein while another might specifically disaggregate tau.

Ultimately, failure of the Hsp110/Hsp70/Hsp40 system might underpin numerous protein-misfolding disorders, and restoration or specific activation of this system might be therapeutically useful (Nillegoda and Bukau, 2015; Shorter, 2016). Indeed, overexpression of Hsp110 with Hsp40 suppressed the toxicity induced by polyglutamine overexpression in *Drosophila*, though it is not apparent if Hsp110 modulates polyglutamine aggregation (Kuo et al., 2013). Additionally, transgenic overexpression of Hsp110 in neurons enhanced survival in ALS model mice, but again, the effects of Hsp110 on SOD1 aggregation were not assessed in these experiments (Nagy et al., 2016). It remains unclear if upregulation of Hsp110 levels will be sufficient to restore normal functionality in animal models, and ultimately in humans. It may be useful to tune the activity of the Hsp110/Hsp70/Hsp40 system using protein-engineering techniques, or alternatively, small-molecule modulators could be developed to enhance the activity of this system. Small heat-shock proteins can also enhance the disaggregase activity of this system (Duennwald et al., 2012), and might also be targeted therapeutically (Makley et al., 2015). However, determining precisely how to therapeutically boost the activity of this system comprised of several components may prove challenging.

## NMNAT

Nicotinamide mononucleotide adenylyl transferases (NMNATs) are nicotinamide adenine dinucleotide (NAD)-synthesizing enzymes. NAD is an important cofactor that mediates numerous cellular processes. NMNATs are important in neuronal maintenance, thus NMNAT knockdown leads to axonal degeneration, while NMNAT overexpression is neuroprotective in several animal models of neurodegeneration (Zhai et al., 2008; Gilley and Coleman, 2010; Ali et al., 2016). NMNAT2 is highly expressed in the mammalian brain, and NMNAT2 mRNA levels are reduced in PD, HD, AD, and tauopathy patients (Ali et al., 2016). Furthermore, elevating NMNAT2 levels in tauopathy model mice suppressed neurodegeneration (Ljungberg et al., 2012). Additionally, NMNAT2 mRNA levels correlate positively with cognitive function and negatively with the pathological features of AD (Ali et al., 2016). In AD brains, NMNAT2 mRNA and protein levels are greatly reduced relative to controls, and NMNAT2 co-localizes with aggregated tau (Ali et al., 2016). NMNAT2 overexpression can reduce the pathological accumulation of hyperphosphorylated tau without altering total tau levels (Ljungberg et al., 2012; Ali et al., 2016). NMNAT2 can prevent protein denaturation and promote protein refolding with similar activity to Hsp70 (Ali et al., 2016). Surprisingly, this activity is maintained even in enzymatically-dead NMNAT2 mutants that lack NAD synthetic activity (Ali et al., 2016). These enzymatically-dead NMNAT2 mutants also reduced hyperphosphorylated tau levels (Ali et al., 2016).

NMNAT2 has been demonstrated to form a complex with Hsp90 to solubilize and refold aggregated substrates (Ali et al., 2016). Moreover, deletion of NMNAT2 increases the vulnerability of cortical neurons to proteotoxic stress (Ali et al., 2016). Thus, therapeutically upregulating NMNAT or enhancing NMNAT activity via small-molecule modulation might be effective in regulating tau levels. It will be interesting to assess the protein-remodeling activity of NMNATs against the many other substrates implicated in protein-misfolding disorders. Given the fundamental role NMNATs play in neuronal maintenance, failure of NMNATs to combat protein misfolding might be common to many other disease-associated substrates in addition to tau.

## HtrA1 can disaggregate and degrade toxic conformers

HtrA1 is a PDZ serine protease that disassembles tau and Aβ fibrils, which are linked to AD, and then degrades them (Poepsel et al., 2015). Intriguingly, HtrA1 is found in the cytoplasm and is also secreted (Poepsel et al., 2015). Correlating with this pattern, Aβ42 fibrils are found in the extracellular space while tau fibrils are found in the cytoplasm. Thus, it has been hypothesized that HtrA1 might be a system that naturally disaggregates and degrades Aβ42 and tau (Poepsel et al., 2015; Shorter, 2016). Indeed, HtrA1 activity might be insufficient in AD patients (Shorter, 2016). Therefore, boosting and fine-tuning the activity of HtrA1 might be valuable in combating AD. It has been demonstrated that HtrA1 activity can be tuned through protein engineering. The disassembly and degradation activities of HtrA1 can be separated, as protease-defective HtrA1 variants dissolve but do not degrade Aβ fibrils, providing HtrA1 variants that can either dissolve the aggregates or dissolve and degrade the aggregates (Poepsel et al., 2015). The ability to separate or combine disassembly and degradation activities in a single protein is very valuable, and might find utility in certain situations. Therefore, it will be very interesting to engineer substrate-specific HtrA1 variants that can target substrates beyond Aβ and tau. These substrate-specific variants could be constructed in both the disaggregate-only or disaggregate-and-degrade backgrounds. This advance would allow for the flexibility to reactivate proteins that serve beneficial functions. Alternatively, subsets of PD patients show increased α-synuclein levels (Ebrahimi-Fakhari et al., 2012), and thus for these patients it may be beneficial to not just solubilize α-synuclein, but also to degrade it. Additionally, small-molecule enhancers of HtrA1 have been identified, and so it will be important to test the effects of these compounds in various models of protein-misfolding disorders (Jo et al., 2014). It will also be important to assess if HtrA1 can clear highly toxic pre-amyloid oligomeric forms of Aβ and tau, or only the fibrils.

## Hsp104 variants suppress protein misfolding, mislocalization, and toxicity in yeast and animal models

Hsp104 is a ring-shaped hexameric AAA+ protein from yeast that serves two distinct functions (Sweeny and Shorter, 2016; Yokom et al., 2016). First, it solubilizes proteins that aggregate following cellular stress to promote yeast survival (Parsell et al., 1991, 1994; Glover and Lindquist, 1998; Glover and Tkach, 2001; Wallace et al., 2015). Second, it regulates yeast prion formation and dissolution (Chernoff et al., 1995; Shorter and Lindquist, 2004, 2005, 2006; Sweeny and Shorter, 2008, 2016; Sweeny et al., 2015). In serving these two roles, Hsp104 recognizes and regulates a diverse milieu of substrates, comprised of the entire yeast proteome, as well as yeast prions (Newby and Lindquist, 2013). While Hsp104 is highly conserved in bacteria, fungi, and plants, Hsp104 has no metazoan homolog (Erives and Fassler, 2015).

The amyloid fold is a highly conserved protein structure, thus it was hypothesized that the natural capacity of Hsp104 to recognize and solubilize yeast prions might translate to a capacity to recognize and solubilize diverse amyloid species associated with human disease (DeSantis et al., 2012; Jackrel and Shorter, 2014b, 2015; Jackrel et al., 2014a). Indeed, using purified proteins, Hsp104 has been shown to solubilize diverse amyloid species implicated in human disease including: Aβ, α-synuclein, polyglutamine expansions, prion protein, tau, and amylin (Liu et al., 2011; DeSantis et al., 2012; Jackrel and Shorter, 2014a; Jackrel et al., 2014a). Additionally, Hsp104 suppresses proteotoxicity in animal models (Satyal et al., 2000; Vacher et al., 2005; Lo Bianco et al., 2008; Cushman-Nick et al., 2013; Jackrel et al., 2014a). In a transgenic mouse model of HD, Hsp104 extended lifespan and decreased aggregate load (Vacher et al., 2005). Furthermore, Hsp104 has been demonstrated to be neuroprotective in a rat model of PD (Lo Bianco et al., 2008). Here, lentiviral vectors coding for α-synuclein were injected into the substantia nigra of rats, and following 6 weeks of expression, brain slices were stained for dopaminergic markers. In this system, Hsp104 co-expression was neuroprotective and no off-target effects were observed (Lo Bianco et al., 2008). Additionally, Hsp104 has been shown to directly clear preformed oligomeric forms of α-synuclein as well as eliminate self-templating α-synuclein conformers. These experiments have provided strong evidence that Hsp104 may have therapeutic value. However, the activity of Hsp104 in suppressing degeneration in these animal models is limited as complete neuroprotection is not achieved (Vacher et al., 2005; Lo Bianco et al., 2008).

We have enhanced the activity of Hsp104 via engineering (Jackrel et al., 2014a,b, 2015; Jackrel and Shorter, 2014a). We have constructed large libraries of randomized Hsp104 variants and developed screening techniques to isolate enhanced variants. When overexpressed in yeast, the proteins TDP-43, FUS, and α-synuclein all form cytoplasmic foci and are toxic (Outeiro and Lindquist, 2003; Johnson et al., 2008; Sun et al., 2011). These yeast models have also empowered the identification of genetic risk factors for these disorders (Elden et al., 2010; Ju et al., 2011; Sun et al., 2011). Deletion or overexpression of Hsp104 does not suppress the toxicity or aggregation of these proteins in yeast (Jackrel et al., 2014a). Thus, these yeast models provide an ideal screening platform to isolate Hsp104 variants with a gain of therapeutic function (Jackrel et al., 2014a,b, 2015). Using these yeast assays, we have identified numerous Hsp104 variants that potently suppress TDP-43, FUS, and α-synuclein toxicity (Jackrel and Shorter, 2014a,b, 2015; Jackrel et al., 2014a,b, 2015). In addition to their suppression of toxicity, these variants also dissolved cytoplasmic foci of TDP-43, FUS, and α-synuclein (Jackrel and Shorter, 2014a; Jackrel et al., 2014a, 2015). Furthermore, the potentiated variants restored alpha-synuclein to the plasma membrance and TDP-43 to the nucleus (Jackrel et al., 2014a). These results are very promising. TDP-43 must shuttle to the nucleus to fulfill its roles in RNA homeostasis, and restoration of nuclear TDP-43 suggests that solubilization of TDP-43 can restore natively folded and functional TDP-43 (Jackrel et al., 2014a). These potentiated Hsp104 variants clear preformed TDP-43, FUS, and α-synuclein fibrils at concentrations where Hsp104 is ineffective (Jackrel et al., 2014a).

To assess the therapeutic utility of potentiated Hsp104 variants, they have been tested in a *C. elegans* model of PD (Jackrel et al., 2014a). Here, the potentiated Hsp104 variants were robustly neuroprotective, while Hsp104 and an ATPase-dead negative control showed no activity (Jackrel et al., 2014a). To further demonstrate the therapeutic possibilities of potentiated Hsp104 variants, it will be essential to demonstrate their activity in additional neuronal models including mammalian neurons. It will also be important to develop additional potentiated Hsp104 variants with improved properties. To do so, it will be crucial to apply additional protein engineering techniques to enhance the activity and substrate specificity of the potentiated variants. There are numerous examples of proteins that are believed to have evolved from roles as generalists to specialists. Thus, it will be very interesting to see if laboratory techniques can accelerate this process for Hsp104 and produce finely-tuned variants. Additionally, perhaps Hsp104 variants can be produced to target pre-amyloid oligomers vs. fibrils and vice versa. In addition to being potentially of direct therapeutic benefit, these variants may hold great value in unraveling the key contributors and drivers of neurodegenerative disease. For instance, engineered disaggregases that can solubilize and reactivate oligomers but not fibrils may be employed as precise mechanistic probes to investigate the effects of resolving specific protein species. Also, the discovery that very subtle modification of natural protein-remodeling factors can confer dramatic alterations in chaperone activity (Jackrel et al., 2014a, 2015) suggests that subtle modification of other protein-remodeling factors, including Hsp110/Hsp70/Hsp40, NMNAT, and HtrA1 might also be amenable to potentiation.

## Conclusions and future directions

Protein misfolding is an enormously challenging issue that underpins many of the most devastating diseases facing society. As the population continues to age, the toll of neurodegenerative disease will continue to rise. Unfortunately, while substantial efforts have been mounted to counter these disorders, there are no treatments available for any of these diseases (with the exception of Tafamidis for FAP). Thus, in the development of new therapeutics to combat these disorders, it will be important to employ innovative approaches. While it is unknown what specifically causes proteins to misfold and cause disease, the accumulation of misfolded aggregates, amyloid, and pre-amyloid species are key contributors to pathogenesis. Therefore, if protein-misfolding trajectories could be reversed, perhaps so could these diseases. Protein-remodeling factors, which have the capacity to block and even reverse protein misfolding might be uniquely positioned as potential therapeutics. Many protein-remodeling factors have been assessed and demonstrated to be potentially useful in combating these disorders. For instance, increased levels or activity of the protein HtrA1 might be employed to dissolve and degrade both tau and Aβ aggregates in AD patients. However, as HtrA1, as well as NMNAT and Hsp110/Hsp70/Hsp40, are all present in humans, it appears that these systems are either insufficient to prevent pathogenesis or are compromised in certain individuals. Thus, it will be important to continue to focus not just on the application of these chaperones directly in disease models, but also to continue to develop approaches to boost and nuance these protein-remodeling systems.

While highly promising, the idea of modulating the proteostasis network is not without caveats. For instance, upregulation of protein-remodeling factors might be beneficial to enhance protein folding and combat neurodegenerative disorders, yet enhanced protein folding might also enable cell proliferation which could promote cancers. Nonetheless, protein-remodeling factors present a unique opportunity to restore proteins to their native fold and function, thus simultaneously alleviating both a loss or gain of function. As with all therapeutics, it will be important to assess for possible off-target effects. For instance, in developing new disaggregase technologies, it will be important to harness protein disaggregation to avoid the unfolding of functional protein complexes. However, it is important to note that Hsp104 does not unfold natively folded proteins. Regardless, it will be important to continue to engineer protein-remodeling factors with desired traits, such as enhanced substrate specificity. New approaches to develop small-molecule modulators of protein-remodeling systems, as well as the engineering of tailored protein-remodeling systems, will prove invaluable in our efforts to rewire and restore the proteostasis network and thus combat neurodegenerative disease.

## Author contributions

MJ and JS wrote and revised the review.

## Funding

MJ is supported by a Target ALS Springboard Fellowship and American Heart Association Postdoctoral Fellowship. JS is supported by the NIH (R01GM099836 and R21NS090205), ALS Association, and the Robert Packard Center for ALS Research at Johns Hopkins.

### Conflict of interest statement

The authors declare that the research was conducted in the absence of any commercial or financial relationships that could be construed as a potential conflict of interest.
